# Temporary, but Essential Requirement of CD8^+^ T Cells Early in the Pathogenesis of Diabetes in BB Rats as Revealed by Thymectomy and CD8 Depletion

**DOI:** 10.1080/10446670310001626508

**Published:** 2003

**Authors:** Herman Groen, Flip Klatter, Jennie Pater, Paul Nieuwenhuis, Jan Rozing

**Affiliations:** Department of Cell Biology Immunology Section Faculty of Medical Sciences University of Groningen Groningen The Netherlands

## Abstract

Autoimmunity-prone BB rats demonstrate a T lymphocytopenia and abnormal T cell subset distribution. To test whether the life span of all T cells or only of certain subsets is reduced in BB rats, we thymectomised 8-week-old BB and PVG rats and subsequently assessed size and composition of the T cell population over a 6-week-period. In both strains, thymectomy (Tx) was followed by a decrease in peripheral T cell numbers, which was proportionally larger in BB rats. The decline of the Thy-1^+^ recent thymic migrant (RTM) T cell phenotype was similar in both strains. BB rats showed a rapid preferential loss of CD8^+^ and CD45RC^+^ T cells, whereas the relative loss of RT6^+^ T cells was proportional to that of all T cells and not significantly different from that in PVG rats. Tx at 8-week did not prevent diabetes. Tx of 4-week-old BB rats revealed essentially the same changes in peripheral T cell subset distribution as in 8-week-old animals. However, Tx at week 4 did prevent diabetes. Since this raised the possibility of a temporary requirement of CD8^+^ T cells for the development of diabetes, we performed CD8 depletions during different pre-diabetic intervals. We found that CD8 depletion from 4 to 8 and 4 to 14 weeks, but not from 8 to 14 weeks of age prevented diabetes. We conclude that the protective effect of early adult Tx is, at least in part, due to the rapid loss of CD8^+^ T cells, and that these cells are only required between 4 and 8 weeks of age for diabetes to develop in BB rats.


*Clinical & Developmental Immunology*, June–December 2003 Vol. 10 (2–4), pp. 141–151

Taylor & Francis healthsciences

### Temporary, but Essential Requirement of CD8$^+$ T Cells Early in the Pathogenesis of Diabetes in BB Rats as Revealed by Thymectomy and CD8 Depletion

HERMAN GROEN*, FLIP KLATTER, JENNIE PATER, PAUL NIEUWENHUIS and JAN ROZING†

*Department of Cell Biology, Immunology Section, Faculty of Medical Sciences, University of Groningen, A. Deusinglaan 1, 9713 AV Groningen, The Netherlands*

Autoimmunity-prone BB rats demonstrate a T lymphocytopenia and abnormal T cell subset distribution. To test whether the life span of all T cells or only of certain subsets is reduced in BB rats, we thymectomised 8-week-old BB and PVG rats and subsequently assessed size and composition of the T cell population over a 6-week-period. In both strains, thymectomy (Tx) was followed by a decrease in peripheral T cell numbers, which was proportionally larger in BB rats. The decline of the Thy-1$^+$ recent thymic migrant (RTM) T cell phenotype was similar in both strains. BB rats showed a rapid preferential loss of CD8$^+$ and CD45RC$^+$ T cells, whereas the relative loss of RT6$^+$ T cells was proportional to that of all T cells and not significantly different from that in PVG rats. Tx at 8-week did not prevent diabetes. Tx of 4-week-old BB rats revealed essentially the same changes in peripheral T cell subset distribution as in 8-week-old animals. However, Tx at week 4 did prevent diabetes. Since this raised the possibility of a temporary requirement of CD8$^+$ T cells for the development of diabetes, we performed CD8 depletions during different pre-diabetic intervals. We found that CD8 depletion from 4 to 8 and 4 to 14 weeks, but not from 8 to 14 weeks of age prevented diabetes. We conclude that the protective effect of early adult Tx is, at least in part, due to the rapid loss of CD8$^+$ T cells, and that these cells are only required between 4 and 8 weeks of age for diabetes to develop in BB rats.

*Keywords*: BB rats; Diabetes; T cell subsets; Thymectomy

### INTRODUCTION

In the rat, different subsets of peripheral T cells can be distinguished from each other by the expression or lack of CD4, CD8, Thy-1 (CD90), RT6 and CD45RC. It has been shown that Thy-1, but not RT6 and CD45RC is expressed by T cells that have recently emigrated from the thymus. These recent thymic migrants (RTM) have been shown to lose the expression of Thy-1 and to acquire RT6 and CD45RC expression after a short period (< 2 weeks) in the periphery (Thiele *et al.*, 1987; Kampinga *et al.*, 1992, 1997; Hosseinzadeh and Goldschneider, 1993).

In the spontaneously diabetic BioBreeding rat, a number of abnormalities has been described regarding the T cell subsets mentioned above, and people have been trying to link these abnormalities to the development of diabetes (Mordes *et al.*, 1996; Greiner *et al.*, 2001; Mordes, 2001). These diabetes-prone BB (DP-BB) rats are severely T lymphocytopenic in that they demonstrate a severely reduced CD4$^+$ T cell population and a virtual lack of CD8$^+$ T cells (Guttmann *et al.*, 1983; Woda *et al.*, 1986; Colle *et al.*, 1992; Mordes, 2001). Nevertheless, CD8$^+$ T cells have been shown to play a crucial role in the development of diabetes in BB rats (Like *et al.*, 1986a,b; Edouard *et al.*, 1993; Ellerman *et al.*, 1993).

An important feature of the lymphocytopenia is the low frequency of RT6$^+$ T cells (Greiner *et al.*, 1986; Bortel *et al.*, 2001; Mordes, 2001; Thiele and Haag, 2001). These cells are known to play a key role in the prevention of diabetes (Mordes *et al.*, 1996). Diabetes-resistant BB (DR-BB) rats that have a very low spontaneous incidence of diabetes (< 1%) and normal numbers of circulating RT6$^+$ T cells develop diabetes upon depletion of these cells (Greiner *et al.*, 1987; Mordes *et al.*, 1996; Mordes, 2001), whereas DP-BB rats can be protected from diabetes by the inoculation of RT6$^+$ T cells (Burstein *et al.*, 1989; Mordes *et al.*, 1996). The frequency of CD45RC$^+$ T cells is also decreased in DP-BB rats (Groen *et al.*, 1989), but to a lesser degree than RT6$^+$ T cells (Groen *et al.*, 1995). Inoculation of CD45RC$^+$ T cells into syngeneic athymic recipients results in pancreatitis, and T cells bearing the CD45RC$^+$ phenotype produce high levels of IL-2 upon *in vitro* stimulation (Mason and Powrie, 1990; Powrie and Mason, 1990). IL-2 is known

*IQ Corporation, Rozenburglaan 13a, 9727 DL Groningen, The Netherlands.
†Corresponding author. Tel.: +31-50-363-2530. Fax: +31-50-363-2512. E-mail: j.rozing@med.rug.nl

ISSN 1740-2522 print/ISSN 1740-2530 online © 2003 Taylor & Francis Ltd
DOI: 10.1080/10446670310001626508

to enhance T cell-mediated islet cytotoxicity (Pukel *et al.*, 1987; O'Shea *et al.*, 2002), whereas anti-IL-2 treatment prevents diabetes in DP-BB rats (Hahn *et al.*, 1987).

Furthermore, absolute numbers of Thy-1$^+$ T cells are reduced in BB rats, whereas frequencies of these cells are increased (Groen *et al.*, 1995; Mordes, 2001). The latter finding indicates that the peripheral T cell pool of pre-diabetic DP-BB rats is less mature than that in age matched control rats. As in normal rats, both RT6 and CD45RC are mainly expressed by mature Thy-1$^-$ T cells (Hosseinzadeh and Goldschneider, 1993; Kampinga *et al.*, 1997), we and others have hypothesised that overall reduction of percentages of RT6$^+$ and CD45RC$^+$ T cells might be a reflection of this relative immaturity in that early cell death might withhold T cells in the DP-BB rat from expressing the RT6 and CD45RC maturation markers (Groen *et al.*, 1989; Crisá *et al.*, 1993; Sarkar *et al.*, 1995). Another possibility is that T cells in the DP-BB rat do reach the Thy-1$^-$/RT6$^+$ or Thy-1$^-$/CD45RC$^+$stages of development, but that the cells bearing these phenotypes have a severely reduced life span, either due to cell death or to change of phenotype.

In the present study, BB and control PVG rats were thymectomized to test whether T cells in the DP-BB rat have a reduced life expectancy in general, or whether different T cell subsets, phenotypically defined by the expression of CD4, CD8, Thy-1, RT6 or CD45RC, have differential life spans. Previously, we have shown that the few CD8$^+$ T cells present in BB rats (Woda *et al.*, 1986) have such a short life span that the vast majority of these cells does not develop to become mature Thy-1$^-$ T cells (Groen *et al.*, 1996a,b). Nevertheless, the role of these few, largely immature CD8$^+$ T cells has been shown to be crucial in the development of IDDM in BB rats, as depletion prevents diabetes (Like *et al.*, 1986a,b; Ellerman *et al.*, 1993), and adoptive transfer of disease depends on their presence (Edouard *et al.*, 1993). Since early adult thymectomy (Tx) has also been shown to prevent the BB diabetic syndrome (Like *et al.*, 1986a,b; Sarkar *et al.*, 1995), we wondered whether this protective effect might lie in a rapid depletion of short-lived CD8$^+$ T cells from the periphery of BB rats. To test this, we assessed whether phenotypic shifts following Tx, with special reference to CD8$^+$ T cells, might help us to understand this protective effect. As our results suggested a temporary requirement for CD8$^+$ T cells in the induction of IDDM, early in the pre-diabetic period, we also performed differential CD8 depletion protocols to test this notion. Our results clearly indicate an association between the protective effect of Tx and a preferential loss of CD8$^+$ and CD45RC$^+$ T cells early in the pre-diabetic period, and an early temporary requirement for CD8$^+$ T cells in the pathogenesis of IDDM in the BB rat.

### MATERIALS AND METHODS

### Rats

Male DP-BB rats were raised and kept under clean conventional conditions at the Central Animal Facility of the University of Groningen, The Netherlands, and had water and food *ad libitum*. Original breeding stock was provided by Organon Pharmaceutics, Oss, The Netherlands. At the time of the study, close to 100% of our colony developed diabetes (>90% between 12 and 20 weeks of age). Male PVG rats were raised under SPF conditions in the Central Animal Laboratory. The University Ethical Board for Animal Studies approved all animal experiments reported in this study.

### Antibodies

The following mAb were used: NDS58 (anti-CD45 (RT7.1)) (Newton *et al.*, 1986), MRC OX-35 (anti-CD4) (Jefferies *et al.*, 1985), MRC OX-8 (anti-CD8) (Brideau *et al.*, 1980), MRC OX-19 (anti-CD5) (Dallman *et al.*, 1984), MRC OX-22 (anti-CD45RC) (Spickett *et al.*, 1983; McCall *et al.*, 1992), ER4 (anti-Thy-1.1) (Vaessen *et al.*, 1985), 3G2 (anti-RT6.1) (Fangmann *et al.*, 1991). Purification and conjugation to FITC and biotin was performed according to standard procedures. As a second step reagent phyco-erythrin conjugated streptavidin (Jackson Laboratories, West Grove, USA) was used.

### Tx and CD8 Depletion

Tx was performed under ether anesthesia by partial sternotomy and surgical resection. Thymi were checked grossly for completeness immediately after the operation, and at autopsy thymic areas were checked both grossly and immunohistochemically. Tx and sham-Tx were performed in PVG rats at 8 weeks and in DP-BB rats at 4, 6 and 8 weeks of age. Depletion of CD8+ T cells was performed with 0.3 mg/kg purified MRC OX-8 Mab given i.p. twice weekly. In the first week of each regimen, rats were treated with three doses.

### Cell Preparation, Labelling and Flow Cytometry

Blood was taken from the tail vein and buffy coats were prepared as previously described (Groen *et al.*, 1995). Cervical lymph nodes (CLN) and spleens were weighed after removal. Suspensions were obtained by exhaustively teasing the tissue through a metal sieve and collecting the cells in cold $Ca^{2+}$ and $Mg^{2+}$ supplemented PBS, containing 5% newborn calf serum (Gibco BRL, Paisley, Scotland) and 0.05% sodium azide. For labeling, each suspension was counted on a Coulter counter (Coulter Epics, Hialeah, USA) and brought to a concentration of approximately $2 \times 10^7$ cells per ml of medium. Labelling and FCM was performed as described previously (Groen *et al.*, 1995).

### Calculation of Absolute Numbers of T Cells and Statistics

For determination of numbers of leukocytes per ml blood, per spleen, and per mg of CLN, two 20 μl samples of unheparinised blood, drawn from the tail vein, and two 20 μl samples of the CLN and spleen cell suspensions described above were counted. Percentages of T cells among leukocytes were established by flow cytometry, using the mAb combination MRC OX-19 (all T cells) plus NDS58 (all leukocytes). From these data, absolute numbers of T cells per unit indicated were calculated for each individual rat. T cell population and subpopulation sizes in rats thymectomised at 8 weeks of age were compared with sham values at 1, 2, 4 and 6 weeks after Tx/sham-Tx and with values obtained at day 0 (Figs. 1 and 2). At 6 weeks after Tx/sham-Tx, one thymectomised DP-BB (TxBB) and one sham-thymectomised DP-BB (sham-TxBB) rat was found diabetic in the last week before sacrifice. Although T cell numbers and subset distribution in these respective rats did not appear different from the other animals in the same group, these were excluded from the results.

Of a small group of DP-BB and PVG rats, T cell (sub)population sizes were determined in blood at day 0 and later at 2 or 4 weeks after Tx ($n = 4$, for each group). This enabled us to calculate reductions in T cell (sub)population sizes within individual DP-BB and PVG rats relative to their own day 0 values (Fig. 3). These relative individual reductions enabled us to test whether there were significant differences in the extent of T cell loss between TxBB and thymectomised PVG (TxPVG) rats, and between different T cell subsets (i.e. CD4 vs CD8). As day 0 and post-Tx values in rats examined twice were not significantly different from those examined only once, these results were pooled, except for Fig. 3, with those of rats sampled only at sacrifice.

All data are presented as means ± SD. Statistical analysis was performed with StatviewTM 5.12$^+$ (Brain-Power Inc, Calabasa, CA), using analysis of variance according to Scheffe. Diabetes incidences were statistically compared using the two-tailed Fisher's exact test.

### Assessment of Diabetes

BB rats were routinely checked for polyuria and polydypsia. From 10 weeks of age, BB rats were checked for glucosuria (Glukotest, Boehringer Mannheim, Manheim, FRG) on a regular basis, and when found postive, blood glucose levels were checked using a Reflolux S haemo-gluco-meter (Boehringer Mannheim). Rast were diagnozed as diabetic when blood glucose levels exceeded 10 mmol/l on two consecutive days. Determination of the diabetes incidence and T cell pool size analyses were performed as two separate experiments using different BB rats.

### RESULTS

### Proportionally Large Short-lived T Cell Population in DP-BB Rats

Figure 1A shows that in DP-BB rats thymectomised at week-8 absolute numbers of T cells per ml blood were significantly decreased compared to sham and day 0 values at all time points after Tx. For PVG rats thymectomised at week 8, the same held from 2 weeks after Tx onward. Sham values remained unchanged as compared to day 0 throughout the experiment. Both in TxBB and TxPVG rats the loss of T cells occurred in the first 2 weeks after Tx, whereafter numbers of T cells more or less stabilised, thus indicating that in both strains the T cell pool consisted of a relatively short-lived and a longer lived T cell population. When relative individual reductions of numbers of total T cells per ml blood were compared in TxBB and TxPVG rats sampled on day 0 and at 2 or 4 weeks after Tx (Fig. 3), it was found that these were significantly larger in TxBB than in TxPVG rats on both post-Tx time points tested.

In CLN of TxBB rats, a mean 35% decrease in numbers of T cells per mg CLN weight was seen after the first week following Tx as compared to sham values, and a mean 50% decrease as compared to day 0 values. At week-2 after Tx and later time points, the respective reductions were approximately 50 and 60%. Numbers of CLN T cells in TxPVG rats demonstrated no significant decrease at 1-week after Tx and 20 and 30% decreases as compared to sham and day 0 values, respectively, from 2 weeks after Tx (data not shown). Numbers of T cells per spleen were determined only at 4 and 6 weeks after Tx and showed no significant differences in TxPVG compared to sham-TxPVG at 4 and 6 weeks after Tx. In TxBB rats, numbers of splenic T cells had significantly decreased approximately 47% at 4 weeks, and 60% at 6 weeks after Tx (data not shown).

Collectively, these findings indicate that the proportion of short lived T cells is significantly larger in DP-BB rats than in PVG rats.

### Loss of most CD8$^+$, but not CD4$^+$ T Cells in the First Week after Tx in DP-BB Rats

As anticipated, the vast majority of CD8$^+$ T cells in TxBB rats disappeared in the first week after Tx (Fig. 1C). The pattern of relative loss of CD4$^+$ T cells was essentially that of total T cells (Fig. 1A and B). In TxPVG rats, on the other hand, the relative loss of CD8$^+$ T cells was similar to that of CD4$^+$ and total T cells (Fig. 1A–C). Figure 3 confirms that there was no significant difference between the relative individual reductions of CD4$^+$, CD8$^+$ and total T cells in TxPVG rats, and between that of CD4$^+$ and total T cells in TxBB rats. CD8$^+$ T cells in TxBB rats, on the other hand, were found to have decreased significantly more when compared to CD4$^+$ and total T cells in TxBB rats at 2 weeks after Tx. At both 2 and 4 weeks after Tx they were significantly more decreased when compared to CD8$^+$ T cells in TxPVG rats. These findings show that, in TxBB rats, most CD8$^+$ T cells have a severely reduced peripheral life span, which is in agreement with what we found in nonthymectomised DP-BB rats (Groen *et al.*, 1996a,b).

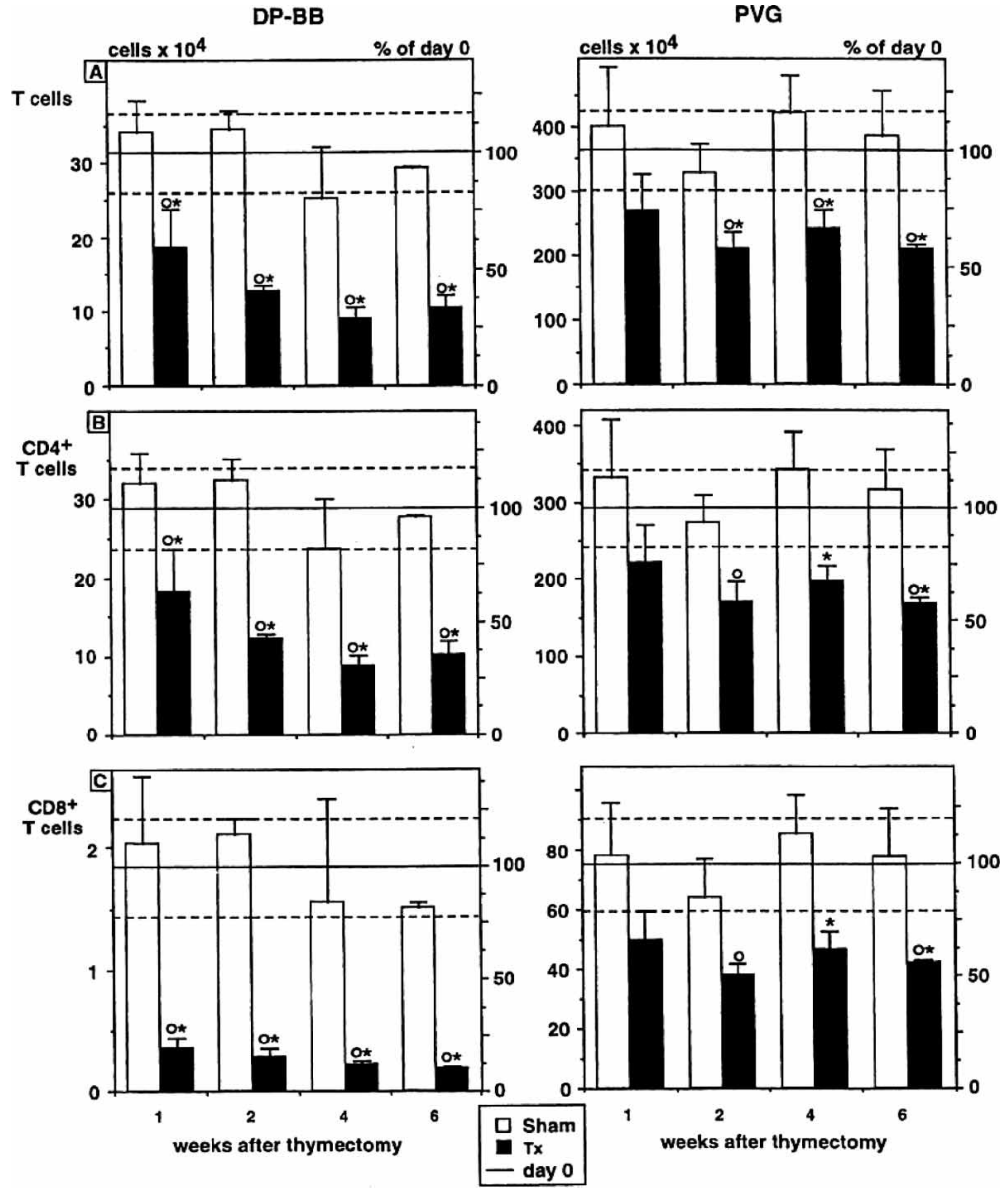

FIGURE 1 Numbers of T cells, $CD4^+$ and $CD8^+$ T cells in thymectomized and sham-thymectomized DP-BB and PVG rats. Absolute numbers ( $\times 10^4$) of (A) T cells, (B) $CD4^+$, and (C) $CD8^+$ T cells per ml of peripheral blood in DP-BB and PVG rats thymectomized or sham-thymectomized at 8 weeks of age. Numbers of rats used were 15 for day 0 and 6–8 on other time points. Bars marked with ° or * indicate a significant difference with day 0 or sham values, respectively.

For $CD8^+$ T cells in CLN of TxBB rats, essentially the same changes were observed, as was also the case for all the subsets following below. For TxPVG rats, detailed subset analysis of CLN T cells was only performed 1 and 2 weeks after Tx. Again, changes were not essentially different from blood (data not shown).

### Similar Decline of the Thy-$1^+$ T Cell Population in TxBB and TxPVG Rats

Table I and Fig. 2A show that percentages and absolute numbers of Thy-$1^+$ T cells rapidly decreased in DP-BB and PVG rats after Tx at 8 weeks and that the relative reductions were similar in the two strains. Statistic comparison of relative individual reductions in DP-BB and PVG rats sampled at day 0 and at 2 or 4 weeks after Tx confirmed that there was no significant difference between the two strains (Fig. 3). This is in agreement with the notion that, like in control rats (Thiele *et al.*, 1987; Kampinga *et al.*, 1992; Hosseinzadeh and Goldschneider, 1993), Thy-1 is a marker for RTM in DP-BB rats (Groen *et al.*, 1995) and shows that the life span of the Thy-$1^+$ phenotype is similar in both strains.

In addition, our results confirm earlier findings that DP-BB rats have a relatively larger, but in absolute size smaller Thy-$1^+$ T cell pool than PVG rats, and that in

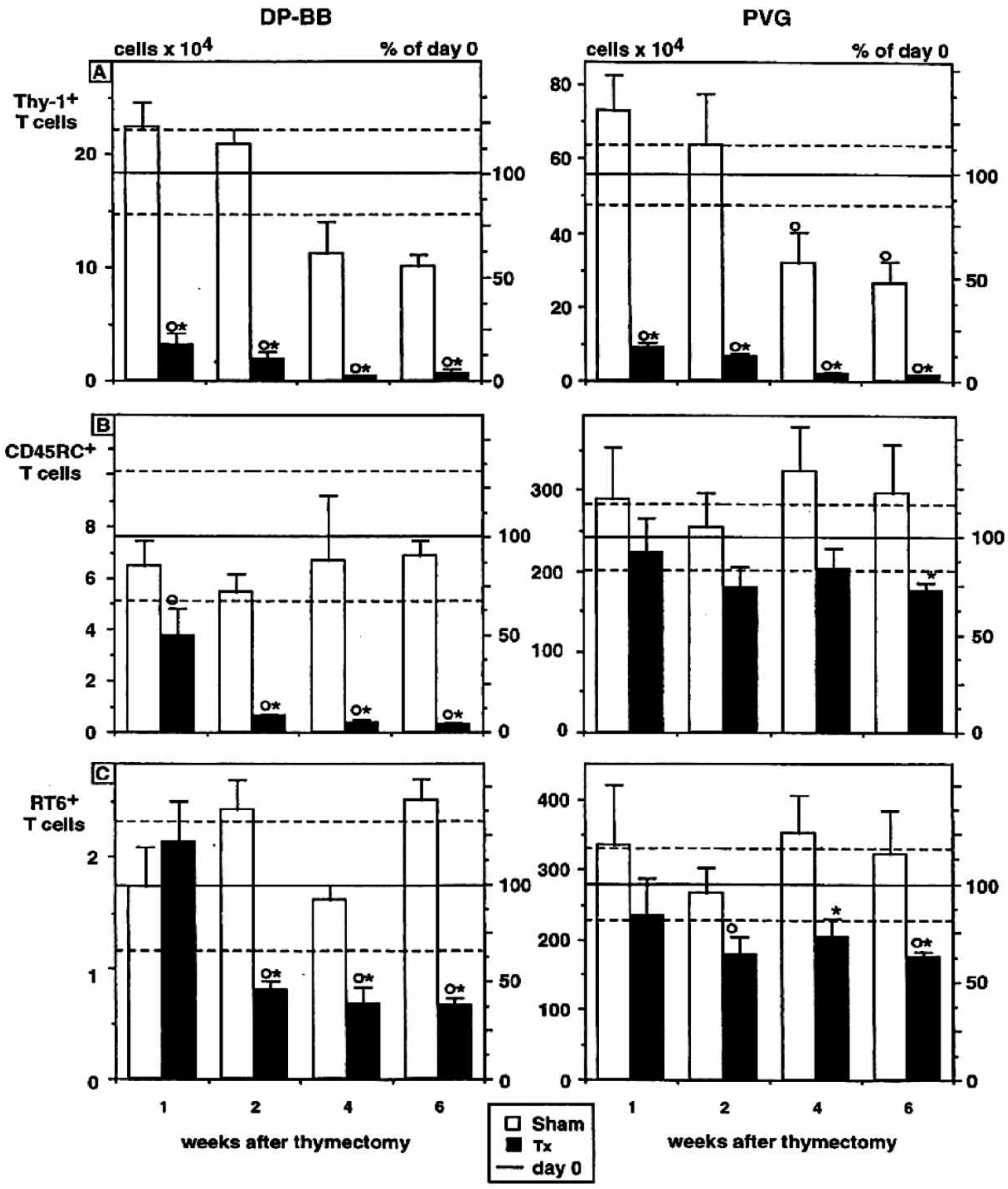

FIGURE 2 Numbers of Thy-1$^+$, CD45RC$^+$ and RT6$^+$ T cells in thymectomised and sham-thymectomised DP-BB and PVG rats. Absolute numbers ( $\times 10^4$) of (A) Thy-1$^+$, (B) CD45RC$^+$, and (C) RT6$^+$ T cells per ml of peripheral blood in DP-BB and PVG rats thymectomised or sham-thymectomised at 8 weeks of age. Numbers of rats used were 15 for day 0 and 6–8 on other time points. Bars marked with ° or * indicate a significant difference with day 0 or sham values, respectively.

both sham-TxBB and sham-TxPVG the population of Thy-1$^+$ T cells decreased with age (Table I, Fig. 2A) (Groen *et al.*, 1995).

### Preferential Loss of CD45RC$^+$ T Cells in TxBB Rats

A substantial reduction in percentages of CD45RC$^+$ T cells, as compared to day 0 and sham values, was noted in TxBB rats from 2 weeks after Tx onward, whereas in TxPVG rats an immediate and persisting increase was noted (Table II). When absolute numbers of CD45RC$^+$ T cells were regarded, a reduction of approximately 50% was already seen during the first week after Tx in DP-BB rats, progressing to an approximate 90% reduction at 2 weeks and later time points after Tx (Fig. 2B). No change in numbers of CD45RC$^+$ T cells could be demonstrated in sham-TxBB rats and in both groups of PVG rats. Figure 3 shows that the relative individual loss of CD45RC$^+$ T cells in TxBB rats was significantly larger than that of total T cells in TxBB rats. These results show that CD45RC$^+$ T cells were preferentially lost in TxBB, but not TxPVG rats.

### No Preferential Loss of RT6$^+$ T Cells in TxBB Rats

Table III shows that in TxPVG rats, percentages of RT6$^+$ T cells increased in the first week after Tx as compared to

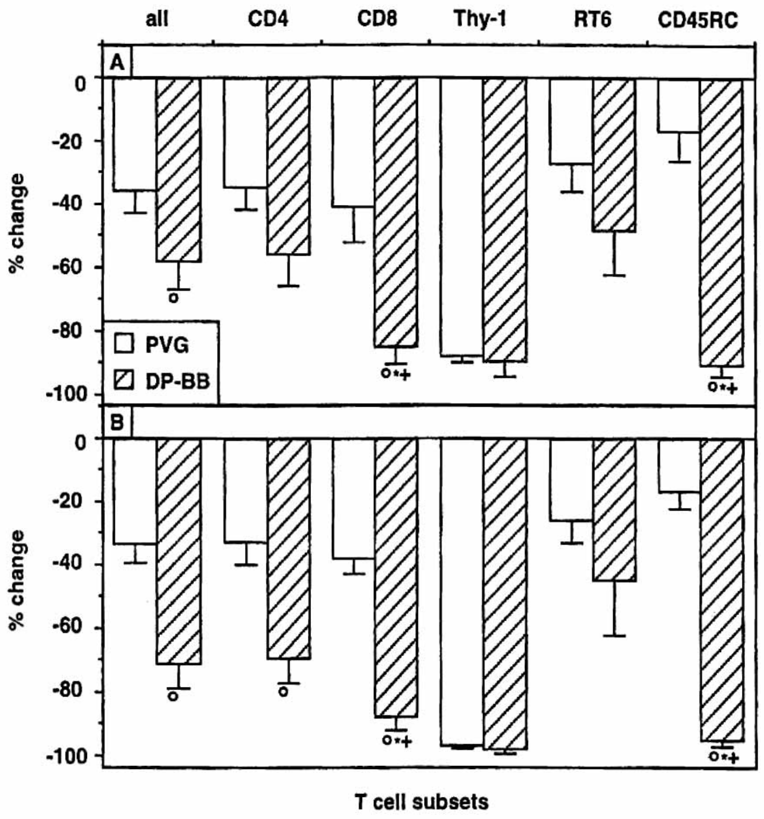

FIGURE 3 Changes in peripheral blood T cell (sub)populations after thymectomy. Peripheral blood T cell (sub)populations were determined in DP-BB and PVG rats at 8 weeks of age (day 0). The rats were thymectomised, and 2 or 4 weeks later these values were determined again in the same rats. Depicted are the means ± SD of the individual relative changes at 2 weeks (A) and 4 weeks (B) after Tx as compared to their own day 0 values. Bars marked with ° indicate a significant differnce between DP-BB and PVG rats ($p < 0.01$). Bars marked with * or + indicate a significant difference as compared to all T cells or $CD4^+$ T cells within the same strain, respectively.

day 0 and from then on remained unchanged, whereas in TxBB rats an initial increase was followed by a decrease to a value not significantly different from day 0. Sham values in both strains (percentages and absolute numbers) remained unaltered as compared to day 0 (Table III, Fig. 2C). At week 1 after Tx, absolute numbers of $RT6^+$ T cells were unchanged in TxBB and TxPVG rats. From then on, these numbers were decreased in both strains. Surprisingly, with respect to relative individual loss of $RT6^+$ T cells at 2 and 4 weeks after Tx, no significant differences were noted between TxBB and TxPVG rats (Fig. 3), which indicates that $RT6^+$ T cells (as a phenotype) are not generally shorter lived in DP-BB than in PVG rats. Furthermore, at 2 weeks after Tx there was no difference between the relative individual loss of $RT6^+$ and total T cells in TxBB rats, and at 4 weeks after Tx the relative individual loss of $RT6^+$ T cells was even significantly less than that of total T cells (Fig. 3). Although percentages and absolute numbers of $RT6^+$ T cells before and after Tx were very low and may have

TABLE I T cells expressing Thy-1 after thymectomy at 8 weeks

| | % Thy-1+ T cells | | | |
|---|---|---|---|---|
| | BB rats | | PVG rats | |
| Time after treatment | Sham | Tx | Sham | Tx |
| Day 0 | 59 ± 5 | | 15 ± 1 | |
| 1 week | 66 ± 2 | 17 ± 1*,** | 19 ± 4 | 3 ± 1*,** |
| 2 week | 61 ± 2 | 15 ± 5*,** | 19 ± 2 | 3 ± 0*,** |
| 4 week | 45 ± 1* | 5 ± 1*,** | 7 ± 1* | 1 ± 0*,** |
| 6 week | 35 ± 1* | 4 ± 2*,** | 7 ± 0* | 1 ± 0*,** |

*Significantly different compared with day 0 ($p < 0.01$).
**Significantly different compared with sham-Tx ($p < 0.01$).

TABLE II T cells expressing CD45RC after thymectomy at 8 weeks

| | % CD45RC+ T cells | | | |
|---|---|---|---|---|
| | BB rats | | PVG rats | |
| Time after treatment | Sham | Tx | Sham | Tx |
| Day 0 | 24 ± 6 | | 66 ± 2 | |
| 1 week | 19 ± 2 | 20 ± 3 | 71 ± 2 | 83 ± 6*,** |
| 2 week | 16 ± 1 | 5 ± 1*,** | 76 ± 4* | 86 ± 2*,** |
| 4 week | 26 ± 6 | 4 ± 1*,** | 76 ± 3* | 83 ± 1*,** |
| 6 week | 23 ± 2 | 3 ± 0*,** | 76 ± 2* | 84 ± 2*,** |

*Significantly different compared with day 0 ($p < 0.01$).
**Significantly different compared with sham-Tx ($p < 0.01$).

TABLE III T cells expressing RT6 after thymectomy at 8 weeks

| Time after treatment | % RT6+ T cells | | | |
|---|---|---|---|---|
| | BB rats | | PVG rats | |
| | Sham | Tx | Sham | Tx |
| Day 0 | 5 ± 1 | | 76 ± 3 | |
| 1 week | 5 ± 1 | 12 ± 2*,** | 82 ± 4 | 87 ± 3* |
| 2 week | 7 ± 1 | 6 ± 0 | 80 ± 2 | 86 ± 2* |
| 4 week | 7 ± 2 | 7 ± 1 | 83 ± 1 | 84 ± 1* |
| 6 week | 9 ± 1 | 6 ± 1 | 82 ± 1 | 84 ± 4 |

*Significantly different compared with day 0 ($p < 0.01$).
**Significantly different compared with sham-Tx ($p < 0.01$).

influenced accuracy, the above suggests that there has been no preferential loss of RT6$^+$ T cells in TxBB rats.

### Complete Protection from Diabetes by Tx at 4, but not 6 and 8 Weeks of Age

Previously, it was found that Tx at 3–5 weeks of age protected DP-BB rats from the onset of diabetes (Like *et al.*, 1986a,b; Sarkar *et al.*, 1995). In our study, DP-BB rats thymectomised at 8 weeks of age (Tx8BB), however, were not protected from diabetes (Table IV). We therefore decided to also thymectomise DP-BB rats at 4 and 6 weeks of age to assess whether a protective effect by Tx in the prediabetic period might be age dependent. In Table IV it is shown that Tx performed at 4 weeks of age (Tx4) was protective, whereas Tx8 was not. Interestingly, Tx at the intermediate timepoint of 6 weeks revealed an intermediate degree of protection in that 3 out of 6 of the animals became diabetic. Diabetic rats in the sham-Tx groups were between 83 and 100%. These findings clearly demonstrate that Tx has to be performed before a critical age, which appears to be earlier than 6 weeks, in order to fully protect DP-BB rats from diabetes. There was no significant difference in age of diabetes onset between TxBB and sham-TxBB rats when checked until 22 weeks (data not shown).

### Protection from Diabetes by Tx is Associated with a Preferential Loss of CD8$^+$ and CD45RC$^+$ T Cells

In Tx8BB rats we saw a rapid and preferential loss of CD8$^+$, Thy-1$^+$, and CD45RC$^+$ T cells (Figs. 1–3). We wondered whether preferential reduction of these subsets could also be demonstrated in rats thymectomised at 4 weeks of age (Tx4BB rats). Furthermore, it was assessed whether at 12 weeks of age, a time point somewhat before the onset of diabetes, differences in subset distribution could be demonstrated between Tx4BB and Tx8BB rats.

TABLE IV Effect of thymectomy on the incidence of diabetes in BB rats

| Thymectomy at | Diabetic rats at 22 weeks | |
|---|---|---|
| | Sham | Tx |
| Week 4 | 5/6 | 0/8* |
| Week 6 | 4/4 | 3/6 |
| Week 8 | 5/5 | 8/8** |

*Significantly different from sham (two-tailed Fisher's exact, 95%, $p = 0.030$).
**Significantly different from Tx4 (two-tailed Fisher's exact, 95%, $p = 0.0002$).

At 2 and 4 weeks after Tx4, DP-BB rats demonstrated a relative decrease of CD8$^+$, Thy-1$^+$, and CD45RC$^+$ T cells as compared to day 0 values and age matched untreated DP-BB rats, whereas percentages of RT6$^+$ T cells remained unchanged (Table V). This is similar to the situation after Tx8 (Tables I–III). At 12 weeks of age, there were also no significant differences between rats thymectomised at 4 or 8 weeks of age. These data suggest a relationship between the induced reduction of one of the aforementioned T cell subsets in a critical period shortly after 4 weeks of age and the protective effect of Tx at 4 weeks. They also show that subset distribution between 4 and 8 weeks of age appears to be of greater imporatnce for the development op diabetes than that just before the age of onset.

### Temporary Requirement of CD8$^+$ T Cells for the Development of Diabetes in DP-BB Rats

The finding that rapid loss of CD8$^+$ T cells after 4 weeks, but not after 8 weeks of age may be associated with protection form IDDM prompted us to study the effect of CD8 depletion from weeks 4 to 8 and from 8 to 14 of age. To test whether in our setting and BB rat colony CD8 depletion would be as effective as described earlier (Like *et al.*, 1986a,b), a group of DP-BB rats was also treated with MRC OX-8 from weeks 4 to 14. Over the same time periods control rats were treated with saline. Since the latter procedure did not influenced development of disease and revealed similar incidences of diabetes in either group, these results were pooled into one saline group (Table VI). We confirmed that CD8 depletion from week 4 to 14 prevented diabetes (Like *et al.*, 1986a,b). Treatment with MRC OX-8 Mab from 4 to 8 weeks resulted in a marked reduction of the diabetes incidence, whereas the incidence in the group treated from 8 to 14 weeks revealed an incidence similar to that in the saline treated group. (Table VI). Lymphocytic insulitis, however, was present in all rats protected from diabetes. CD4$^-$CD8$^+$ T cells were completely absent from the blood 1 week after the first injection with MRC OX-8. One week after the last injection percentages of CD8$^+$ T cells were similar to those in saline treated animals (data not shown).

These results demonstrate that, for the processes leading to disease, CD8$^+$ T cells are only required in the early pre-diabetic life of DP-BB rats, whereas their absence after 8 weeks of age does not appear to have consequences for the development of diabetes.

### DISCUSSION

### Changes in the T Cell Population of Thymectomised Rats

In the first part of this study we investigated whether the T lymphocytopenia in DP-BB rats is due to a general or

TABLE V T cell subset distribution in CLN of non-Tx- and Tx-BB rats from 4 to 8 weeks, and in 12 weeks old DP-BB rats thymectomized at 4 or 8 weeks

| Age (week) when | | % of T cells expressing | | | | |
|---|---|---|---|---|---|---|
| Tx | FACS* | CD4 | CD8 | Thy-1 | RT6 | CD45RC |
| – | 4 | 95 ± 0 | 5 ± 1 | 70 ± 1 | 7 ± 2 | 23 ± 3 |
| – | 6 | 94 ± 1 | 5 ± 1 | 55 ± 2** | 7 ± 2 | 19 ± 3 |
| – | 8 | 96 ± 2 | 3 ± 0 | 39 ± 4**,‡ | 3 ± 0 | 16 ± 2 |
| 4 | 6 | 97 ± 1 | 2 ± 1**,† | 10 ± 2**,† | 7 ± 2 | 7 ± 1**,† |
| 4 | 8 | 99 ± 0** | 0 ± 0**,† | 4 ± 0**,† | 2 ± 0** | 6 ± 0**,† |
| 4 | 12 | 99 ± 1** | 1 ± 0** | 2 ± 0** | 2 ± 0** | 7 ± 2** |
| 8 | 10 | 99 ± 1** | 1 ± 0** | 2 ± 0** | 3 ± 1** | 7 ± 2** |
| 8 | 12 | 99 ± 0** | 1 ± 0** | 3 ± 1** | 3 ± 1** | 6 ± 1** |

*$n = 4–5$. Statistic comparison was performed within the same subset between day 0 (week4, non-Tx) and all other values.
**$p < 0.01$, between Tx and age-matched non-Tx rats.
†$p < 0.01$, between week 6 and week 8 non-Tx values.
‡$p < 0.01$, and between week 12 Tx4 and week 12 Tx8 values (no significant differences).

to a non-randomly reduced life span of peripheral T cell subsets. It was reported earlier that adult Tx segregates a short lived, directly thymus dependent T cell population from a longer lived T cell population which persists without the presence of a thymus (Kappler *et al.*, 1974; Berzins *et al.*, 1998; Cunningham *et al.*, 2001; Lee *et al.*, 2001). Such segregation between short and longer lived T cells is clearly demonstrated for the TxBB and TxPVG rats investigated in the present study (Fig. 1A). In TxBB rats, however, the short lived T cell population was found to be proportionally larger than that in TxPVG rats (Fig. 3), which is likely to indicate that also in untreated DP-BB rats, a proportionally larger part of the T cell population than in PVG rats will be short lived, and will contribute to the T lymphocytopenia. This is largely in line with earlier proposals of a reduced life span for T cells in DP-BB rats (Like *et al.*, 1986a,b; Groen *et al.*, 1989; 1996a,b; Sarkar *et al.*, 1995), however, it clearly does not hold for all T cells in these rats.

We have shown earlier, that the vast majority of CD8$^+$ T cells in DP-BB rats has such a short life span that the cells do not reach the Thy-1$^-$ compartment of T cell maturation (Groen *et al.*, 1996a,b). In the present study this was confirmed by the loss of ~90% of CD8$^+$ T cells in TxBB rats within 1 week after Tx (Fig. 1C). Although the mechanism underlying this severely reduced life expectancy still remains to be resolved, it adds to the explanation for a near total absence of CD8$^+$ T cells in DP-BB rats (Woda *et al.*, 1986).

The rapid and severe decrease of Thy-1$^+$ T cells after Tx in DP-BB and PVG rats (Table I, Figs. 2A and 3) confirms earlier findings that Thy-1$^+$ T cells are RTM which disappear rapidly after Tx (Kampinga *et al.*, 1992; Hosseinzadeh and Goldschneider, 1993; Groen *et al.*, 1995). The disappearance of Thy-1$^+$ T cells may either be due to maturational transformation to a Thy-1$^-$ phenotype, to loss of Thy-1$^+$ T cells by cell death, or to a mixture of both. The earlier finding that the T cell population in DP-BB rats already substantially decreased in size during the Thy-1$^+$ stage of peripheral maturation (Groen *et al.*, 1996a,b) suggests that transition of Thy-1$^+$ T cells to the mature Thy-1$^-$ stage of development is not as efficient in DP-BB rats as it is in control rats. Therefore, although RTM reduction rates were found to be similar in TxBB and TxPVG rats, kinetics may not be. We believe it reasonable to assume that the relative contribution of early cell death to the Tx-induced loss of Thy-1$^+$ T cells is larger in DP-BB rats than it is in PVG rats. Based on their experiments, Zadeh and Goldschneider concluded likewise (Zadeh *et al.*, 1996).

Similar to CD8$^+$ T cells, approximately 90% of CD45RC$^+$ T cells was rapidly lost in TxBB, but not TxPVG rats (Table II, Fig. 2B), probably indicating that in untreated DP-BB rats CD45RC$^+$ T cells (as a phenotype) have a short life span. Since we have earlier reported that acquisition of CD45RC on immature Thy-1$^+$ T cells is not disturbed in DP-BB rats (Groen *et al.*, 1996a,b), the short life span of the CD45RC$^+$ phenotype may be the major contributory factor to the reduced frequency of CD45RC$^+$ T cells observed in DP-BB rats. However, whether the reduction in phenotypical CD45RC$^+$ T cells is caused by actual loss of cells or change of phenotype cannot be concluded from the present study.

Somewhat surprizingly, we found RT6$^+$ T cells in TxBB rats to disappear proportionally to the reduction of the total T cell population and to RT6$^+$ T cells in TxPVG rats (Table III, Figs. 2C and 3). It appears that, once T cells in the DP-BB rat have reached the RT6$^+$ stage of development, a normal proportion of these cells remains RT6$^+$. This indicates that the RT6$^+$ T cell phenotype in DP-BB rats may not have a reduced life span, but that the low frequency of RT6$^+$ T cells observed in DP-BB rats is better explained by a decreased input from the preceding

TABLE VI Effect on the incidence of diabetes in BB rats treated with MRC OX-8 over different time periods

| CD8 depletion | Diabetic rats | % diabetic |
|---|---|---|
| Saline | 9/11 | 82 |
| 4–8 weeks | 4/16* | 25 |
| 8–14 weeks | 12/16 | 75 |
| 4–14 weeks | 0/5* | 0 |

*Significantly different from saline and 8–14 weeks group (two-tailed Fisher's exact, 95%, $p \leq 0.01$).

RT6$^-$ stage of development. This may be either due to disturbed acquisition of RT6 expression on developing T cells, or to major loss of cells in the maturational pathway leading to RT6 expression. The latter two defects have been reported to occur in DP-BB rats (Angelillo *et al.*, 1988; Groen *et al.*, 1996a,b; Mordes, 2001; Thiele and Haag, 2001).

Taken together, our results show that Tx-induced loss of the short lived T cell population in DP-BB rats is not randomly distributed among the subsets investigated. Tx in DP-BB rats selectively affects the Thy-1$^+$, the CD8$^+$ and the CD45RC$^+$ T cell subsets, of which the latter two were earlier found to be largely contained within the Thy-1$^+$ subset. These data also suggest an inefficient transition from Thy-1$^+$ to Thy-1$^-$ T cells in DP-BB rats.

**T Cell Subset Distribution and Diabetes in DP-BB Rats**

In the second part of this study, it was assessed whether the protective effect of Tx in DP-BB rats with respect to the development of diabetes as demonstrated by others (Like *et al.*, 1986a,b; Sarkar *et al.*, 1995) was reflected in changes in the peripheral T cell subset distribution.

Prevention of diabetes by Tx (Like *et al.*, 1986a,b; Sarkar *et al.*, 1995) and by CD8 depletion (Like *et al.*, 1986a,b; Ellerman *et al.*, 1993), and a short lifespan for CD8$^+$ T cells in BB rats (Groen *et al.*, 1996a,b) raised the question of whether a rapid loss of CD8$^+$ T cells following Tx might be underlying the protective effect of Tx. Indeed, CD8$^+$ T cells were found to die rapidly after Tx at 8 weeks. However, this could not be linked directly to a protective effect since Tx at 8 weeks did not prevent diabetes. As Tx at 4 weeks did prevent diabetes, while having a similar effect on the peripheral T cell pool as Tx at 8 weeks, we wondered whether the presence of CD8$^+$ T cells might only be important with respect to the onset of diabetes in the early pre-diabetic life of DP-BB rats and no longer at 8 weeks of age. Our differential CD8$^+$ T cell depletions clearly show that this is indeed the case in DP-BB rats.

The reduction of CD4$^+$/CD45RC$^+$ T cells after Tx may also be important in the prevention of diabetes as these cells have been reported to produce high levels of the Th1 associated cytokines IL-2 and $\gamma$-IFN upon stimulation, and to result in pancreatitis upon injection into syngeneic nude rats (Mason and Powrie, 1990; Powrie and Mason, 1990). IL-2 has been shown to enhance islet cytotoxicity (Pukel *et al.*, 1987; O'Shea *et al.*, 2002), whereas anti-IL2 treatment prevent diabetes in DP-BB rats (Hahn *et al.*, 1987). The possibility that CD4$^+$/CD45RC$^+$ T cells are essential helper/inducer T cells for the generation of cytotoxic CD8$^+$ T cells cannot be excluded, and is presently being investigated by treatment with anti-CD45RC antibodies.

The period between 4 and 8 weeks of age clearly is a critical period with respect to the eventual development of diabetes in DP-BB rats. A similar critical period has been demonstrated earlier both for the prevention of diabetes in DP-BB rats and for the induction of diabetes in RT6 depleted diabetes-resistant (DR) BB rats (Greiner *et al.*, 1987; Burstein *et al.*, 1989). These studies demonstrate that BB rats can become diabetic even when RT6$^+$ T cells are present, provided that they were absent around 4 weeks of age, and also that DR-BB rats are protected from diabetes in the absence of RT6$^+$ T cells when these were present around 4 weeks of age (Mordes *et al.*, 1996; Mordes, 2001). Our study adds to this knowledge the observation that even in the virtual absence of RT6$^+$ T cells (in DP-BB rats) around the age of 4 weeks, Tx or depletion of CD8$^+$ T cells, when performed at that time, can prevent disease, but less effective, or not at all, when performed later in life. These results taken together indicate that either removal by Tx and CD8 depletion, or suppression by RT6$^+$ T cells of autoreactive CD8$^+$ T cells in DP-BB rats is only effective when occurring long before the onset of diabetes.

Why does removal of CD8$^+$ T cells by 4 weeks of age result in complete prevention of diabetes, whereas removal of these cells is less protective or not at all, at 6 and 8 weeks of age, respectively? Although we and others (Voorbij *et al.*, 1989; Mordes, 2001) have never observed massive lymphocytic insulitis in DP-BB rats under 8 weeks of age, some activated autoreactive CD8$^+$ T cells may have already infiltrated and damaged some of the islets at young age. Removal of such autoreactive T cells (Like *et al.*, 1986a,b; Ellerman *et al.*, 1993) or grafting of regulatory RT6$^+$ T cells (Burstein *et al.*, 1989; Mordes *et al.*, 1996) at the right time would then prevent initial damage and hence diabetes. We have shown earlier that thymocyte subset distribution and reduced expression of CD8 on pre-selection thymocytes in DP-BB rats indeed suggest the generation of autoreactive CD8$^+$ T cells that escape negative selection (Groen *et al.*, 1996a,b). In fact, it has been shown that thymocytes of DR-BB rats have the potency to cause diabetes in athymic recipient rats after RT6 depletion (Whalen *et al.*, 1995; 2001).

Also changes in islet physiology and antigenicity specific for the period from 4 to 8 weeks of age could make the islets more vulnerable for immunological attack at that stage. Rats are normally weaned between 21 and 28 days of age. This event marks a number of important changes in the rat's physiology. In the context of beta-cell vulnerablity, one of the most important changes is the switch from mother milk to solid food. It has been shown that basic insulin levels before weaning are much lower than afterwards (Pierzynowski *et al.*, 1993) and, in addition, after weaning a food challenge will result in a beta-cell insulin response, whereas before weaning only glucose levels will rise post-prandially, but not insulin levels (Penicaud *et al.*, 1991; Pierzynowski *et al.*, 1993; 1995; Knapper *et al.*, 1995) the enhanced insulin response mentioned is related to protein content of the food taken (Zetterstrom *et al.*, 1985). Insulin secretion increases the expression of several islet cell antigens (Appel *et al.*, 1989; Roep *et al.*, 1990; McCulloch *et al.*, 1991) and beta-cells have been shown to be more susceptible to *in vitro* cellular immune destruction when they are glucose stimulated than when they are not (Ekblond *et al.*, 1997). In addition, numbers of lymphocytes are known to peak 2 weeks after weaning (McCauley and Hartmann, 1984). Therefore, it is highly likely that the beginning of the critical 4–8 weeks window is caused by an increased beta-cell activity (on a per-cell basis) in conjunction with increasing numbers of (autoreactive) lymphocytes. Another change around the time of weaning is a massive proliferation of pancreatic cells (Johnson *et al.*, 1980; Vidal *et al.*, 1994). This change may be more endogenously regulated than food-induced, since the proliferation of pancreatic cells has been repeatedly observed from approximately 1 week before the food switch until 2 weeks after (Rodriguez-Martin *et al.*, 1996). This pancreatic mitotic activity might also contribute to increased beta-cell vulnerability to cellular immune destruction (Ekblond *et al.*, 1997).

Moreover, dietary changes upon weaning may also influence the T cell system directly. Colonization of the gut takes place in early life (Simecka, 1998). Also changing from mother's milk to a regular infant formula is associated with major changes in the spectrum of intestinal bacteria species (Harmsen *et al.*, 2000). Extensive "cross-talk" between the intestinal flora and the mucosal immune system seems to exist, since Jiang *et al.* (2001) have shown that the developing immune system itself influences the establishment of the gut flora. The intestinal flora therefore seem to play a key role in establishing (mucosal) immunity and tolerance (Simecka, 1998; Hooper *et al.*, 2001). It might be interesting to note that both DP-BB and DR-BB rats display a limited V-β TCR usage until around week-6, whereafter DR-BB rats, but not DP-BB rats show a remarkable expansion (Gold and Bellgrau, 1991). This expansion might rescue the DR-BB rat from becoming diabetic.

### *Acknowledgements*

We thank Drs M. Newton (Oxford, UK), K. Wonigeit (Hannover, FRG), and Dr D. Mason (Oxford, UK) for the kind donation of mAb producing cell lines, and Dr H. Verheul and E. Cremers (Organon Pharmaceutics, Oss, The Netherlands) for the donation of the original BB/O breeding nucleus. This work was supported by a grant of the Dutch Diabetes Foundation (DFN96.606).

### *References*

Angelillo, M., Greiner, D.L., Mordes, J.P., Handler, E.S., Nakamura, N., McKeever, U. and Rossini, A. (1988) "Absence of RT6$^+$ T cells in diabetes-prone BioBreeding/Worcester rats is due to genetic and cell developmental defects", *J. Immunol.* **141**, 4146–4151.

Appel, M.C., Dotta, F., O'Neil, J.J. and Palmer, J.P. (1989) "B-cell activity regulates the expression of islet antigenic determinants", *Diabetologia* **32**, 461A.

Berzins, S.P., Boyd, R.L. and Miller, J.F. (1998) "The role of the thymus and recent thymic migrants in the maintenance of the adult peripheral lymphocyte pool", *J. Exp. Med.* **187**, 1839–1848.

Bortel, R., Waite, D.J., Whalen, B.J., Todd, D., Leif, J.H., Lesma, E., Moss, J., Mordes, J.P., Rossini, A.A. and Greiner, D.L. (2001) "Levels of Art2+ cells but not soluble Art2 protein correlate with expression of autoimmune diabetes in the BB rat", *Autoimmunity* **33**, 199–211.

Brideau, R.P., Carter, P.B., McMaster, W.R., Mason, D.W. and Williams, A.F. (1980) "Two subsets of rat T lymphocytes defined with monoclonal antibodies", *Eur. J. Immunol.* **10**, 609–615.

Burstein, D., Mordes, J.P., Greiner, D.L., Stein, D., Nakamura, N., Handler, E.S. and Rossini, A.A. (1989) "Prevention of diabetes in BB/Wor rat by single transfusion of spleen cells. Parameters that affect degree of protection", *Diabetes* **38**, 24–30.

Colle, E., Fuks, A., Poussier, P., Edouard, P. and Guttmann, R.D. (1992) "Polygenic nature of spontaneous diabetes in the rat. Permissive MHC haplotype and presence of the lymphopenic trait of the BB rat are not sufficient to produce susceptibility", *Diabetes* **41**, 1617–1623.

Crisá, L., Sarkar, P., Waite, D.J., Haag, F., Koch-Nolte, F., Rajan, T.V., Mordes, J.P., Handler, E.S., Thiele, H.G. and Rossini, A.A. (1993) "An RT6$^a$ gene is transcribed and translated in lymphopenic diabetes-prone BB rats", *Diabetes* **42**, 688–695.

Cunningham, C.P., Kimpton, W.G., Fernando, A. and Cahill, R.N. (2001) "Neonatal thymectomy identifies two major pools of sessile and recirculating peripheral T cells which appear to be under separate homeostatic control", *Int. Immunol.* **13**, 1351–1359.

Dallman, M.J., Thomas, M.J. and Green, J.R. (1984) "MRC OX-19, a monoclonal antibody that labels rat T lymphocytes and augments *in vitro* proliferative response", *Eur. J. Immunol.* **14**, 260–267.

Edouard, P., Hiserodt, J.C., Plamondon, C. and Poussier, P. (1993) "CD8$^+$ T-cells are required for adoptive transfer of the BB rat diabetic syndrome", *Diabetes* **42**, 390–397.

Ekblond, A., Schou, M. and Buschard, K. (1997) "Mononuclear cytotoxicity and proliferation towards glucose stimulated rodent pancreatic islet cells", *Autoimmunity* **25**, 97–108.

Ellerman, K., Wrobleski, M., Rabinovitch, A. and Like, A.A. (1993) "Natural killer cell depletion and diabetes mellitus in the BB/Wor rat (revisited)", *Diabetologia* **36**, 596–601.

Fangmann, J., Schwinzer, R., Hedrich, H-J., Klöting, I. and Wonigeit, K. (1991) "Diabetes-prone BB rats express the RT6 alloantigen on intestinal intraepithelial lymphocytes", *Eur. J. Immunol.* **21**, 2011–2015.

Gold, D.P. and Bellgrau, D. (1991) "Identification of a limited T-cell receptor β chain variable region repertoire associated with diabetes in the BB rat", *Proc. Natl Acad. Sci.* **88**, 9888–9891.

Greiner, D.L., Handler, E.S., Nakano, K., Mordes, J.P. and Rossini, A.A. (1986) "Absence of the RT6 T cell subset in diabetes-prone BB/W rats", *J. Immunol.* **136**, 148–151.

Greiner, D.L., Mordes, J.P., Handler, E.S., Angelillo, M., Nakamura, N. and Rossini, A.A. (1987) "Depletion of RT6$^+$ T lymphocytes induces diabetes in resistant BioBreeding (BB/W) rats", *J. Exp. Med.* **166**, 461–475.

Greiner, D.L., Rossini, A.A. and Mordes, J.P. (2001) "Translating data from animal models into methods for preventing human autoimmune diabetes mellitus: caveat emptor and primum non nocere", *Clin. Immunol.* **100**, 134–143.

Groen, H., van der Berk, J.M.M.M., Nieuwenhuis, P. and Kampinga, J. (1989) "Peripheral T cells in diabetes prone (DP) BB rats are CD45R-negative", *Thymus* **14**, 145–150.

Groen, H., Klatter, F.A., Brons, N.H.C., Wubbena, A.S., Nieuwenhuis, P. and Kampinga, J. (1995) "High-frequency, but reduced absolute numbers of recent thymic migrants among peripheral blood T lymphocytes in diabetes-prone BB rats", *Cell. Immunol.* **163**, 113–119.

Groen, H., Klatter, F.A., Brons, N.H.C., Mesander, G., Nieuwenhuis, P. and Kampinga, J. (1996) "Abnormal thymocyte subset distribution and differential reduction of CD4$^+$ and CD8$^+$ T cell subsets during peripheral maturation in Diabetes-prone BB rats", *J. Immunol.* **156**, 1269–1275.

Groen, H., Pater, J.M., Klatter, F.A., Nieuwenhuis, P. and Rozing, J. (1996) "Expression of RT6, but not CD45RC is disturbed on immature peripheral T cells in the BB rat", *Adv. Exp. Med. Biol.* **419**, 253–256.

Guttmann, R.D., Colle, E., Michel, F. and Seemayer, T. (1983) "Spontaneous diabetes mellitus syndrome in the rat. II. T lymphopenia and its association with clinical disease and pancreatic lymphocytic infiltration", *J. Immunol.* **130**, 1732–1735.

Hahn, H.J., Lucke, S., Klöting, I., Volk, H.D., Baehr, R.V. and Diamantstein, T. (1987) "Curing BB rats of freshly manifested diabetes by short-term treatment with a combination of a monoclonal anti-interleukin 2 receptor antibody and a subtherapeutic dose of cyclosporin A", *Eur. J. Immunol.* **17**, 1075–1078.

Harmsen, H.J., Wildeboer-Veloo, A.C., Raangs, G.C., Wagendorp, A.A., Klijn, N., Bindels, J.G. and Welling, G.W. (2000) "Analysis of intestinal flora development in breast-fed and formula-fed infants by using molecular identification and detection methods", *J. Pediatr. Gastroentrol. Nutr.* **30**, 61–67.

Hooper, L.V., Wong, M.H., Thelin, A., Hansson, L., Falk, P.G. and Gordon, J.I. (2001) "Molecular analysis of commensal host-microbial relationships in the intestine", *Science* **291**, 881–884.

Hosseinzadeh, H. and Goldschneider, I. (1993) "Recent thymic emigrants in the rat express a unique pheno-type and undergo post-thymic maturation in peripheral tissues", *J. Immunol.* **150**, 1670–1679.

Jefferies, W.A., Green, J.R. and Williams, A.F. (1985) "Authentic T helper CD4 (W3/25) antigen on rat peritoneal macrophages", *J. Exp. Med.* **162**, 117–127.

Jiang, H.Q., Bos, N.A. and Cebra, J.J. (2001) "The timing, localization and persistence of colonization by segmented filamentous bacteria in the neonatal mouse gut depend on immune status of mothers and pups", *Inf. Immunity* **69**, 3611–3617.

Johnson, L.R., Dudrick, S.J. and Guthrie, P.D. (1980) "Stimulation of pancreatic growth by intraduodenal amino acids and HCl", *Am. J. Physiol.* **239**, 400–405.

Kampinga, J., Groen, H., Klatter, F.A., Meedendorp, B., Aspinall, R., Roser, B. and Nieuwenhuis, P. (1992) "Postthymic T cell development in rats: An update", *Biochem. Soc. Trans.* **20**, 191–197.

Kampinga, J., Groen, H., Klatter, F.A., Pater, J.M., van Petersen, A.S., Roser, B. and Nieuwenhuis, P. (1997) "Post-thymic T cell development in the rat", *Thymus* **24**, 173–200.

Kappler, J.W., Hunter, P.C., Jacobs, D. and Lord, E. (1974) "Functional heterogeneity among the T-derived lymphocytes of the mouse. I. Analysis by adult thymectomy", *J. Immunol.* **113**, 27–38.

Knapper, J.M., Morgan, L.M., Fletcher, J.M. and Marks, V. (1995) "Plasma and intestinal concentrations of GIP and GLP-1 (7–36) amide during suckling and after weaning in pigs", *Horm. Metab. Res.* **27**, 485–490.

Lee, C.K., Kim, K., Welniak, L.A., Murphy, W.J., Muegge, K. and Durum, S.K. (2001) "Thymic emigrants isolated by a new method possess unique phenotypic and functional properties", *Blood* **97**, 1360–1369.

Like, A.A., Biron, C.A., Weringer, E.J., Byman, K., Srocyski, E. and Guberski, D.L. (1986) "Prevention of diabetes in BioBreeding/Worcester rats with monoclonal antibodies that recognize T lymphocytes or natural killer cells", *J. Exp. Med.* **164**, 1145–1159.

Like, A.A., McGill, P.D. and Sroczynski, E. (1986) "Adult thymectomy prevents diabetes mellitus in BB/W rats", *Diabetologia* **29**, 565A.

Mason, D. and Powrie, F. (1990) "Memory $CD4^+$ T cells in man form two distinct subpopulations, defined by their expression of isoforms of the leucocyte common antigen, CD45", *Immunology* **70**, 427–433.

McCall, M.N., Shotton, D.M. and Barclay, A.N. (1992) "Expression of soluble isoforms of rat CD45. Analysis by electron microscopy, and use in epitope mapping of anti-CD45R monoclonal antibodies", *Immunology* **76**, 310–317.

McCauley, I. and Hartmann, P.E. (1984) "Changes in piglet leucocytes B lymphocytes and plasma cortisol from birth to three weeks after weaning", *Res. Vet. Sci.* **37**, 234–241.

McCulloch, D.K., Barmeier, H., Neifing, J.L. and Palmer, J.P. (1991) "Metabolic state of the pancreas affects end-point titre in the islet cell antibody assay", *Diabetologia* **34**, 622–625.

Mordes, J.P. (2001) "Autoimmune diabetes in the BB rat", Primer on Animal Models of Diabetes (Harwood Academic Publishers, Amsterdam), pp. 1–41.

Mordes, J.P., Bortell, R., Doukas, J., Rigby, M.R., Whalen, B.J., Zipris, D., Greiner, D.L. and Rossini, A.A. (1996) "The BB/Wor rat and the balance hypothesis of autoimmunity", *Diabetes/Metab. Rev.* **2**, 103–109.

Newton, M.R., Wood, K.J. and Fabre, J.W. (1986) "A monoclonal alloantibody detecting a polymorphism of the rat leucocyte common (LC) antigen", *J. Immunogenet.* **13**, 41–50.

O'Shea, J.J., Ma, A. and Lipsky, P. (2002) "Cytokines and autoimmunity", *Nat. Rev. Immunol.* **2**, 37–45.

Penicaud, L., Ferre, P., Assimacopoulos-Jeannet, F., Perdereau, D., Leturque, A., Jeanrenaud, B., Picon, L. and Girard, J. (1991) "Increased gene expression of lipogenic enzymes and glucose transporter in white adipose tissue of suckling and weaned obese Zucker rats", *Biochem. J.* **279**, 303–308.

Pierzynowski, S.G., Westrom, B.R., Erlanson-Albertsson, C., Ahre'n, B., Svendsen, J. and Karlsson, B.W. (1993) "Induction of exocrine pancreas maturation at weaning in young developing pigs", *J. Pediatr. Gastroenterol. Nutr.* **16**, 287–293.

Pierzynowski, S.G., Westrom, B.R., Svendsen, J., Svendsen, L. and Karlsson, B.W. (1995) "Development and regulation of porcine pancreatic function", *Int. J. Pancreatol.* **18**, 81–94.

Powrie, F. and Mason, D. (1990) "OX-$22^{high}$ $CD4^+$ T cells induce wasting disease with multiple organ pathology: Prevention by the OX-$22^{low}$ subset", *J. Exp. Med.* **172**, 1701–1708.

Pukel, C., Baquerizo, H. and Rabinovitch, A. (1987) "Interleukin 2 activates BB/W diabetic rat lymphoid cells cytotoxic to islet cells", *Diabetes* **36**, 1217–1222.

Rodriguez-Martin, E., Munoz-Acedo, G., Garcia-Escribano, C., Rodriguez-Puyol, M. and Arilla, E. (1996) "Lactational changes in the rat exocrine pancreas somatostatin receptors and modulation of guanylate cyclase", *Biochim. Biophys. Acta* **1316**, 102–108.

Roep, B.O., Arden, S.D., de Vries, R.R. and Hutton, J.C. (1990) "T-cell clones from a type-1 diabetes patient respond to insulin secretory granule proteins", *Nature* **345**, 632–634.

Sarkar, P., Crisá, L., McKeever, U., Bortell, R., Handler, E., Mordes, J.P., Waite, D., Schoenbaum, A., Haag, F. and Koch-Nolte, F. (1995) "Loss of RT6 message and most circulating T cells after thymectomy of diabetes-prone BB rats", *Autoimmunity* **18**, 15–22.

Simecka, J.W. (1998) "Mucosal immunity of the gastrointestinal tract and oral tolerance", *Adv. Drug. Deliv. Rev.* **34**, 235–259.

Spickett, G.P., Brandon, M.R., Mason, D.W., Williams, A.F. and Woollett, G.R. (1983) "MRC OX-22, a monoclonal antibody that labels a new subset of T lymphocytes and reacts with the high molecular weight form of the leukocyte-common antigen", *J. Exp. Med.* **158**, 795–810.

Thiele, H.G. and Haag, F. (2001) "The RT6 system of the rat: developmental, molecular and functional aspects", *Immunol. Rev.* **184**, 96–108.

Thiele, H-G., Koch, F. and Kashan, A. (1987) "Postnatal distribution profiles of Thy-$1^+$ and $RT6^+$ cells in peripheral lymph nodes of DA rats", *Transpl. Proc.* **19**, 3157–3160.

Vaessen, L.M.B., Joling, P., Tielen, F.J. and Rozing, J. (1985) "New surface antigens on T cells in the early phase of T cell differentiation, detected by monoclonal antibodies", *Adv. Exp. Med. Biol.* **186**, 251–254.

Vidal, C., Rauly, I., Zeggari, M., Delesque, N., Esteve, J.P., Saint-Laurent, N., Vaysse, N. and Susini, C. (1994) "Up-regulation of somatostatin receptors by epidermal growth factor and gastrin in pancreatic cancer cells", *Mol. Pharmacol.* **46**, 97–104.

Voorbij, H.A.M., Jeucken, P.H.M., Kabel, P.J., de Haan, M. and Drexhage, H.A. (1989) "Dendritic cells and scavenger macrophages in pancreatic islets of prediabetic BB rats", *Diabetologia* **38**, 1623–1629.

Whalen, B.J., Marounek, J., Weiser, P., Appel, M.C., Greiner, D.L., Mordes, J.P. and Rossini, A.A. (2001) "BB rat thymocytes cultured in the presence of islets lose their ability to transfer autoimmune diabetes", *Diabetes* **50**, 972–979.

Whalen, B.J., Rossini, A.A., Mordes, J.P. and Greiner, D.L. (1995) "DR-BB rat thymus contains thymocyte populations predisposed to autoreactivity", *Dev. Biol.* **44**, 963–967.

Woda, B.A., Like, A.A., Padden, C. and McFadden, M.L. (1986) "Deficiency of phenotypic cytotoxic-suppressor T lymphocytes in the BB rat", *J. Immunol.* **136**, 856–859.

Zadeh, H.H., Greiner, D.L., Wu, D.Y., Tausche, F. and Goldschneider, I. (1996) "Abnormalities in the export and fate of recent thymic emigrants in diabetes-prone BB/W rats", *Autoimmunity* **24**, 35–46.

Zetterstrom, R., Ginsburg, B.E., Lindblad, B.S. and Persson, B. (1985) "Relation between protein intake, plasma valine, and insulin secretion during early infancy", *Klin. Padiatr.* **197**, 371–374.

